# Galectin-3: an early predictive biomarker of modulation of airway remodeling in patients with severe asthma treated with omalizumab for 36 months

**DOI:** 10.1186/s13601-017-0143-1

**Published:** 2017-03-09

**Authors:** Anna Maria Riccio, Pierluigi Mauri, Laura De Ferrari, Rossana Rossi, Dario Di Silvestre, Louise Benazzi, Alessandra Chiappori, Roberto Walter Dal Negro, Claudio Micheletto, Giorgio Walter Canonica

**Affiliations:** 1 Respiratory Diseases and Allergy Unit, IRCCS AOU San Martino-IST, University of Genoa, Genoa, Italy; 2 Proteomics and Metabolomics Unit, Institute for Biomedical Technologies, CNR, Milan, Italy; 3 National Centre for Respiratory Pharmacoeconomics and Pharmacoepidemiology, CESFAR, Verona, Italy; 4 Respiratory Unit, Mater Salutis Hospital, Legnago, Verona Italy; 5 Department of Biomedical Sciences, Personalized Medicine Clinic Asthma and Allergy, Humanitas University, Rozzano, Milan, Italy

**Keywords:** Anti-IgE, Omalizumab, Severe asthma, Galectin-3, Biomarker, Airway remodeling, Bronchial biopsy, Proteomics, Eosinophils

## Abstract

**Background:**

Bronchial asthma is a heterogeneous disease characterized by three cardinal features: chronic inflammation, variable airflow obstruction, and airway hyperresponsiveness. Asthma has traditionally been defined using nonspecific clinical and physiologic variables that encompass multiple phenotypes and are treated with nonspecific anti-inflammatory therapies. Based on the modulation of airway remodeling after 12 months of anti-immunoglobulin E (IgE) treatment, we identified two phenotypes (omalizumab responder, OR; and non-omalizumab responder, NOR) and performed morphometric analysis of bronchial biopsy specimens. We also found that these two phenotypes were correlated with the presence/absence of galectin-3 (Gal-3) at baseline (i.e., before treatment). The aims of the present study were to investigate the histological and molecular effects of long-term treatment (36 months) with anti-IgE and to analyze the behavior of OR and NOR patients.

**Methods:**

All patients were treated with the monoclonal antibody anti-IgE omalizumab for 36 months. The bronchial biopsy specimens were evaluated using morphometric, eosinophilic, and proteomic analysis (MudPIT). New data were compared with previous data, and unsupervised cluster analysis of protein profiles was performed.

**Results:**

After 36 months of treatment with omalizumab, reduction of reticular basement membrane (RBM) thickness was confirmed in OR patients (Gal-3-positive at baseline); similarly, the protein profiles (over 500 proteins identified) revealed that, in the OR group, levels of proteins specifically related to fibrosis and inflammation (e.g., smooth muscle and extracellular matrix proteins (including periostin), Gal-3, and keratins decreased by between 5- and 50-fold. Eosinophil levels were consistent with molecular data and decreased by about tenfold less in ORs and increased by twofold to tenfold more in NORs. This tendency was confirmed (p < 0.05) based on both fold change and DAVE algorithms, thus indicating a clear response to anti-IgE treatment in Gal-3-positive patients.

**Conclusions:**

Our results showed that omalizumab can be considered a disease-modifying treatment in OR. The proteomic signatures confirmed the presence of Gal-3 at baseline to be a biomarker of long-term reduction in bronchial RBM thickness, eosinophilic inflammation, and muscular and fibrotic components in omalizumab-treated patients with severe asthma. Our findings suggest a possible relationship between Gal-3 positivity and improved pulmonary function.

**Electronic supplementary material:**

The online version of this article (doi:10.1186/s13601-017-0143-1) contains supplementary material, which is available to authorized users.

## Background

Bronchial asthma is a heterogeneous disease characterized by three cardinal features: chronic inflammation, variable airflow obstruction, and airway hyperresponsiveness [1]. The heterogeneity of asthma extends beyond clinical symptoms, response to therapy, age at onset, duration of disease process, extent of bronchial narrowing, sensitivity to triggering agents, airway inflammatory pattern, and the immune response. Accordingly, classifying asthma into several smaller, more homogeneous subgroups, the so-called phenotypes, makes it easier to define the underlying mechanisms of the disease, administer more effective mechanism-based therapy, and improve prediction of disease course.

Technological advances during the last decade have facilitated clinical and molecular research, thus making it possible to combine phenotyping approaches to optimize asthma management and personalized medicine [2].

The US Severe Asthma Research Program (SARP) recently analyzed an extensive dataset of patients with severe and nonsevere asthma in order to identify and describe robust subgroups of asthma patients with specific features [3] that could guide personalized therapy, although this approach has not yet been verified. Using a variety of statistical analyses, the authors interrogated the dataset to identify the characteristics that most accurately distinguish between subgroups within the study population. This approach is known as clustering [1].

Asthma has traditionally been defined using nonspecific clinical and physiological variables that encompass multiple phenotypes and has been treated using nonspecific anti-inflammatory therapies. Recent molecular and genetic studies have identified clinical and inflammatory phenotypes associated with specific biomarkers.

Biomarkers for inflammation driven by type-2 helper T lymphocytes (Th2), including elevated fractional exhaled nitric oxide (FeNO) levels, blood/sputum eosinophil counts, and serum periostin levels, have helped to identify a Th2-high molecular phenotype of asthma. Treatment of Th2-high asthmatic patients with biologic agents targeting immunoglobulin E (IgE) and the canonical Th2-related cytokines interleukin (IL) 4, IL-5, and IL-13 is proving to be efficacious [4]. However, we do not yet know the biomarkers able to identify a Th2-low asthma phenotype and potentially guide therapy. Although targeted biologic agents are generally efficacious in treating various phenotypes of asthma and allergic disease, some patients respond better to one biologic agent than another or not at all. The reasons for these differential responses are still unknown [5].

We performed morphometric analysis on bronchial biopsy specimens before and after anti-IgE treatment to investigate modulation (or not) of airway remodeling after 12 months of treatment with omalizumab. We identified two phenotypes of severe asthma: the omalizumab responder phenotype (OR) and the non-omalizumab responder phenotype (NOR) [6]. Proteomic analysis of the specimens showed that these patient subgroups were characterized by different levels of galectin-3 (Gal-3) in bronchial tissue at baseline and after 12 months of treatment with omalizumab [7]. In the present study, we extended our morphometric, eosinophilic, and proteomic analyses by investigating bronchial biopsy specimens collected from the same patients after 36 months of treatment with omalizumab and compared molecular data with data on bronchial reticular basement membrane (RBM) thickness and bronchial eosinophilic and neutrophilic infiltration.

The aims of the present study were to evaluate proteomic signatures and modifications in RBM thickness with respect to long-term anti-IgE treatment and to investigate the behaviour of OR and NOR.

## Methods

### Patients

Eight patients were treated with the monoclonal antibody anti-IgE omalizumab for 36 months. The patients’ clinical features (Table 1) were described in a previous paper, where we showed that the original RBM thickness and eosinophil infiltration were reduced in a substantial proportion of severe asthmatics after 1 year of treatment with omalizumab, thus emphasizing the possible role of this agent in airway remodelling in severe persistent allergic asthma [6].

**Table 1:** Patient characteristics

Patients (n)	8
Age (years)	47.0 ± 9.7
Sex ratio (M/F)	5/3
Smokers/nonsmokers	1/7
Body mass index	23.8 ± 3.1
Total plasma IgE (kU/L)	309.4 ± 218.2
Forced expiratory volume in the first second	52% pred ± 14%
Asthma control test score	11.3 ± 2.8

Data are expressed as mean ± SEM

The study was approved by the Ethics Committee of Orlandi General Hospital of Bussolengo, Verona, Italy.

### Bronchial biopsy: collection and processing

As previously reported before treatment and at 12 months [6], each patient underwent bronchoscopy 36 months after starting anti-IgE treatment. Bronchial biopsy specimens were obtained using a flexible bronchoscope (Pentax FB19-TX, Langley, UK). Specimens were collected from the right middle lobe, fixed in formalin for 10 h at 4 °C, and embedded in paraffin. The blocks were cut into 3-µm sections using a rotary microtome.

### Morphometric analysis

The bronchial sections were mounted onto glass slides, dewaxed, rehydrated, and stained with hematoxylin-eosin (Hematoxylin and Eosin Stain, Carl Roth GmbH + Co. KG, Germany).

The area of the RBM was measured using computer-aided digital morphometry with a DFC 320 Leica color digital camera attached to a Leica Microsystems DMLA light microscope. Digital images of the biopsies were captured at high power using a 100× lens and analyzed using QWin software (Leica Microsystems). Bronchial RBM thickness was measured according to the recommendations of the American Thoracic Society/European Respiratory Society using the orthogonal intercept method. A 100 × 100-μm grid was randomly overlaid on hematoxylin-eosin-stained sections. Orthogonal intercepts were measured from the intersections of the grid with the RBM-epithelium junction to the RBM-subepithelial area junction. At least 40 measures were obtained every 20 μm. Morphometric analysis was performed by two blinded operators. The arithmetic means of the intercept measures were calculated using the formula *τ* = *π/*4 × *arithmetic mean of orthogonal intercepts* [8].

### Evaluation of the eosinophilic infiltrate

Bronchial biopsies collected 36 months after starting treatment with omalizumab were considered suitable for examination if at least 1.0 mm of RBM length and 0.1 mm^2^ of subepithelial area were morphometrically preserved. Eosinophils were quantified in the area extending 50 μm under the RBM and expressed as number of eosinophils/mm^2^ of subepithelium (mean of two samples per patient). The eosinophil count was performed by two blinded operators [9].

### Evaluation of the neutrophilic infiltrate

In order to obtain complete data on the bronchial inflammatory pattern, we evaluated neutrophilic infiltration before treatment and after 36 months. This variable was not investigated in our previous paper [6]. Biopsies were considered suitable for examination when there was at least 1.0 mm of RBM length and 0.1 mm^2^ of subepithelial area. Neutrophils were quantified in the area extending 50 μm under the RBM and expressed as number of neutrophils/mm^2^ of subepithelium. The neutrophil count was performed by two blinded operators [9].

### Proteomic analysis

The dewaxed tissues (3-µm section from each sample) were dried in a vacuum centrifuge and resuspended in 0.1 M ammonium bicarbonate, pH 7.9. Proteins were extracted from tissue as previously described [7]. Briefly, tissue was homogenized in solution buffer (0.1 M ammonium bicarbonate, pH 7.9) treated with RapiGest SF reagent (Waters Corporation, Milford, MA, USA) and incubated with stirring, first at 100 °C for 20 min and then at 80 °C for 2 h. Subsequently, the protein concentration was assayed using the SPN^TM^ Protein Assay kit (G-Biosciences, Maryland Heights, MO, USA), and 5 ± 0.5 µg of protein from each sample was digested with trypsin (Sequencing Grade Modified Trypsin, Promega, Madison, WI, USA) using a 1:50 (w/w) enzyme/substrate ratio at 37 °C overnight. The next morning, an additional aliquot of enzyme was added at an enzyme/substrate ratio of 1:100 (w/w), and digestion was continued for 4 h. Enzyme digestion was stopped by addition of trifluoroacetic acid to reach a pH of 2, and the digested samples were desalted and enriched using PepClean columns (Pierce Biotechnology, Rockford, IL, USA).

The resulting peptide mixtures were analyzed using multidimensional protein identification technology (MudPIT) [10] based on two-dimensional chromatography coupled to tandem mass spectrometry (2DC-MS/MS). Peptides were identified to correlate the experimental tandem mass spectra with the theoretical peptide sequences obtained by the in silico digestion of a human protein database (approximately 230,000 entries) downloaded from the NCBI website (www.ncbi.nlm.nih.gov).

### Statistical and clustering analyses

Mass spectra data were processed using Bioworks version 3.3.1 based on the SEQUEST algorithm (University of Washington, licensed to Thermo Finnigan Corp., San José, CA, USA) and the following parameters: Xcorr scores greater than 1.5 for singly charged peptide ions and 2.0 and 2.5 for doubly and triply charged ions, respectively; peptide probability ≤0.001; and protein consensus score value ≥10. These filters guaranteed that the resulting proteins had a p value of ≤0.001. The false-positive peptide ratio, which was calculated through the reverse database, was less than 3%.

The statistical analysis was performed using R software. Biological and technical replicates were evaluated by hierarchical clustering [11] using an in-house R script based on the XlsReadWrite, clue, and clValid libraries (http://cran.r-project.org). The Euclidean distance metric was applied and an agglomerative coefficient was calculated.

We measured five parameters (eosinophils, smooth muscle proteins, periostin, keratins, and RBM) in eight patients (OR, n = 4; NOR, n = 4) and separated them according to time (T0, and T36) to evaluate significant differences between OR and NOR. For each group, significant differences were also evaluated between baseline (T0) and T36.

As for eosinophils (cells/mm^2^), the *t* test (p ≤ 0.05) was used to evaluate the difference between the patient groups (OR and NOR) and treatment time (T0 and T36). The same comparisons were performed for periostin, Gal-3, smooth muscle proteins, and keratins. In this case, the average SEQUEST score values were evaluated using the Wilcoxon test because proteomic data are not normally distributed (Shapiro–Wilk test).

Variation in the proteins analyzed was also evaluated by calculating the fold change, as previously reported [12]; this was defined as the natural logarithm of the ratio T36/T0. In addition, the protein lists were analyzed and aligned using multidimensional algorithm protein map software (MAProMa), which provides quantitative indexes based on the SEQUEST score, such as DAVE and DCI [13]. DAVE is an index of the ratio between the two conditions compared and, therefore, indicates different amounts of each protein under the two different conditions. When the protein is not present in the reference (baseline), DAVE is +2.0, whereas if the protein is not present in the sample (in our case after treatment, T36), DAVE is −2.0. Consequently, intermediate DAVE values indicate different amounts of protein under the two conditions compared. DAVE values >|0.4| indicate significant variations. Finally, the *t* test was used to evaluate significant differences in the average DAVE and fold change values.

## Results

We evaluated the effect of long-term anti-IgE treatment (36 months) on RBM thickness, eosinophilic and neutrophilic infiltrates, and proteomic profiles by analyzing bronchial biopsy specimens from patients with severe asthma (Table 1).

### Histological analysis

All bronchial biopsies were evaluated using morphometric analysis of RBM thickness.

Differences were observed in the behavior of the two groups (OR and NOR) between baseline and 36 months of anti-IgE treatment.

**Fig. 1:**
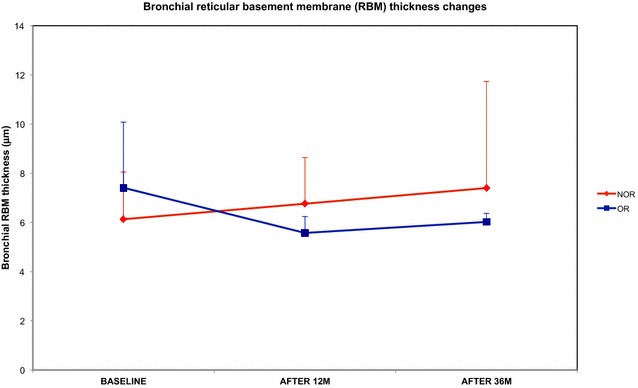
Changes in bronchial RBM thickness in omalizumab responders (OR) and non-omalizumab responders (NOR) at baseline and after 12 and 36 months of anti-IgE treatment

Analysis of bronchial tissue in the OR patients revealed a consistent reduction in RBM thickness; conversely, analysis of the NOR patients revealed an increase in RBM thickness despite treatment with omalizumab (Fig. 1).

**Fig. 2:**
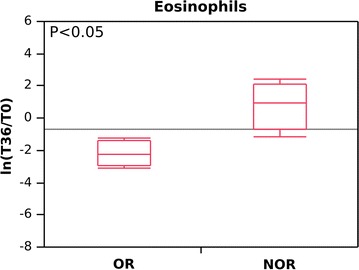
Evaluation of bronchial eosinophilic infiltration (cells/mm^2^) in omalizumab responders (OR) and non-omalizumab responders (NOR) using the natural logarithm of the score fold change, (ln[T36/T0]). Negative values indicate a decrease in the eosinophil count at T36; positive values indicate an increase in the eosinophil count at T36. OR and NOR are classified according to whether they experienced a reduction in RBM thickness (OR) or no reduction in RBM thickness (NOR) after 12 months of anti-IgE treatment

As for subepithelial eosinophils, our data showed that the eosinophilic infiltrate doubled (7–15/mm^2^) in NOR patients after 36 months. Analysis of biopsy specimens from the OR patients, on the other hand, revealed the presence of eosinophils to be about 10 times lower (55–5/mm^2^) (Fig. 2). The difference between OR and NOR was significant (p < 0.05; fold change = ln[T36/T0]) (Additional file 1).

Evaluation of the neutrophilic infiltrate revealed no differences between the two groups. Moreover, the number of neutrophils in bronchial specimens at baseline, after both 12 months and 36 months was very low and negligible (data not shown).

## Protein profile

MudPIT made it possible to identify 546 distinct proteins (see Additional file 2 for a complete list of the proteins identified for each sample). Using the MAProMa software, each protein list was automatically plotted onto a 2D map according to the theoretical molecular weight (MW) and isoelectric point (pI) of the proteins identified. A representative example is reported in Additional file 3, which shows the 2D maps corresponding to all the proteins identified, thus confirming the possibility of characterizing proteins with extreme theoretical MW values (<10 kDa) and proteins with extreme pI values (<4 or >10).

In our previous study [6], the main differences at 12 months affected the cytoskeleton, mainly smooth muscle and keratins, and ECM proteins. With respect to ECM proteins, analysis of the biopsy specimens at 36 months revealed the same trend of proteomic signature for patients who were Gal-3-positive before treatment (OR); in contrast, protein behavior was different in patients who were Gal-3-negative before treatment (NOR).

**Fig. 3:**
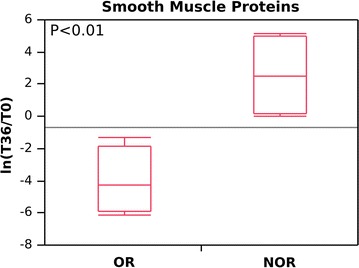
Changes in abundance levels, expressed as the natural logarithm of the score fold change (ln[T36/T0]) for smooth muscle proteins (SMPs) in omalizumab responders (OR) and non-omalizumab responders (NOR) at baseline (T0) and after 36 months (T36) of treatment. A negative value indicates a decrease in SMPs at T36; a positive value indicates an increase in SMPs at T36. OR and NOR are classified according to whether they experienced a reduction in RBM thickness (OR) or no reduction in RBM thickness (NOR) after 12 months of anti-IgE treatment

In the OR group, smooth muscle proteins (including myosins, tropomyosins, and actins) had decreased to very low levels at 36 months (from 265 to 8 [aggregate score]; average ln[T36/T0], about –4.0; DAVE, –1.89) in the OR group (Gal-3-positive biopsies). In the NOR group (Gal-3-negative biopsies), protein levels increased from 5 to 74 (average ln[T36/T0], around +2.5; DAVE, +1.75) (Fig. 3; Additional files 1, 4). The difference between the two groups was almost significant (p < 0.01).

**Fig. 4:**
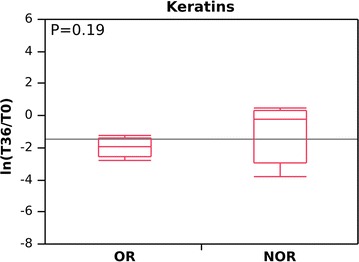
Changes in abundance levels, expressed as the natural logarithm of the score fold change (ln[T36/T0]) for keratins in omalizumab responders (OR) and non-omalizumab responders (NOR) at baseline (T0) and after 36 months (T36). A negative value indicates a decrease in keratins at T36; a positive value indicates an increase in keratins at T36. In this case, the p value is high; however, if NOR2 is excluded, the p value falls to 0.002 (Additional file 1). OR and NOR are classified according to whether they experienced a reduction in RBM thickness (OR) or no reduction in RBM thickness (NOR) after 12 months of anti-IgE treatment

As for keratins, the trend at 36 months of treatment was more consistent for OR (Gal-3 positive) (about sixfold less, from 1800 to 300 as the average score; average ln[T36/T0], −2; DAVE −1.45) than NOR (both DAVE and average ln[T36/T0], low stringent). In this case the difference was not significant (p = 0.19; see Fig. 4; Additional files 1, 4).

**Fig. 5:**
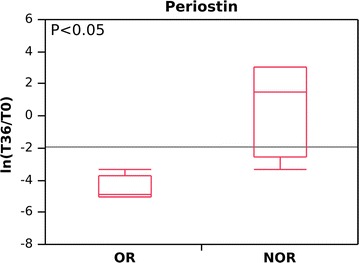
Changes in abundance levels expressed as the natural logarithm of the score fold change (ln[T36/T0]) for periostin in omalizumab responders (OR) and non-omalizumab responders (NOR) at baseline (T0) and after 36 months (T36) of anti-IgE treatment. A negative value indicates a decrease in periostin at T36; a positive value indicates an increase in periostin at T36. OR and NOR are classified according to whether they experienced a reduction in RBM thickness (OR) or no reduction in RBM thickness (NOR) after 12 months of anti-IgE treatment

ECM proteins, which were present almost exclusively at baseline in OR (Gal-3-positive) patients, also maintained the decreasing trend at 36 months. Specifically, periostin was reduced in all OR patients (from 117 to 0 average score; average ln[T36/T0], −4.6; DAVE −2.0), whereas in the NOR group, it was either stable (NOR3) or increased (NOR1 and NOR4), except for NOR2, in whom the periostin level decreased. Figure 5 shows the behavior (ln[T36/T0]) of periostin in OR and NOR over time (p < 0.05; see also Additional files 1, 4).

Gal-3 was stable at 12 months, but was absent in OR at 36 months (Gal-3-positive at T0). Gal-3 was not detectable at 36 months in NOR (Gal-3-negative at T0) (Additional file 5).

As reported above, NOR patients, who were characterized as Gal-3-negative at T0 (before treatment), presented the same behavior after 36 months of treatment; however, the trend for one patient (NOR2) after 36 months of treatment was more similar to that of OR than NOR patients for each of the main parameters monitored. In fact, in NOR2, both smooth muscle proteins and keratins had decreased or were absent at 36 months, as was the case for OR patients. Similarly, eosinophils and periostin were, respectively, reduced and absent in NOR2, as in OR and in contrast to the remaining NOR.

Table 2 summarizes the levels of the main protein classes and eosinophils before treatment (T0) and 36 months after treatment for each OR and NOR sample. Of note, both the ln[T36/T0] (Additional file 1) and DAVE index (Additional file 6) confirm the different trends for OR and NOR in the parameters examined at baseline and T36. They also highlight the different behavior of NOR2 at T36.

**Table 2:** Levels of eosinophils (cells/mm^2^) and abundance (score) for smooth muscle proteins, periostin, and keratins at baseline (T0) and after 36 months of anti-IgE treatment (T36)

	OR1	OR2	OR3	OR4	NOR1	NOR2	NOR3	NOR4
Eosinophils								
T0	19	31	58	115	0	10	10	9
T36	3	9	4	5	*11*	3	*20*	*27*
Smooth muscle proteins								
T0	288	80	198	494	0	0	20	0
T36	10	20	0	0	*168*	0	*30*	*98*
Periostin								
T0	30	160	130	150	0	30	120	0
T36	0	0	0	0	*20*	0	*106*	*20*
Keratins								
T0	1990	1902	1630	2047	1404	1910	1892	1676
T36	574	276	100	266	*2106*	40	1330	*1400*
Galectin-3								
T0	10	10	30	20	0	0	0	0
T36	0	0	0	0	0	0	0	0

Increased values (or substantially unchanged) at T36 compared to T0 are reported in italic. For more details about available proteins see Additional file 2

## Clustering analysis

**Fig. 6:**
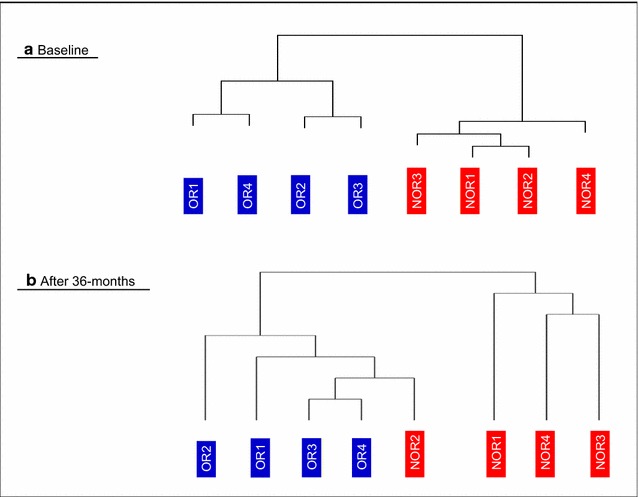
Unsupervised hierarchical clustering of protein lists obtained by analyzing lung biopsies from omalizumab responders (OR, *blue*) and non-omalizumab responders (NOR, *red*). **a** Before (baseline). **b** After 36 months of anti-IgE treatment. The clusters obtained were based on SEQUEST score value and proved to be well structured (agglomerative coefficients >0.7). OR and NOR are classified according to whether they experienced a reduction in RBM thickness (OR) or no reduction in RBM thickness (NOR) after 12 months of anti-IgE treatment

Protein lists from the 36-month biopsy specimens were compared and used to perform an unsupervised clustering analysis. Thus, OR (Gal-3-positive), who were correctly segregated at T0 (see Fig. 6a), were again confirmed to be part of the same group at 36 months. It is noteworthy that patient NOR2 was in this group. In addition, the remaining three NOR patients (NOR1, NOR3, and NOR4, all Gal-3-negative) were segregated in a different cluster (see Fig. 6b), thus confirming them to be part of the same NOR group again at 36 months of treatment. These findings confirm the different behavior at 36 months of patient NOR2, who was segregated with the OR group (see Table 2).

## Discussion

Biomarkers of severe asthma are currently a major research area [14]. Nonetheless, available biomarkers [15] are still far from enabling us to select appropriate candidates for biologics [16, 17]. The need for molecular analysis to define phenotypes has been discussed [18], and omics sciences have been proposed as a useful approach [19].

In this context, current advances in proteomics, mainly those based on mass spectrometry, facilitate the discovery-driven studies of new biomarkers in respiratory diseases and improve the clinical reliability of biomarkers for asthma [20].

The main focus of the present study was the extension of the histological and proteomic analyses to 36 months in a group of patients with severe asthma treated with omalizumab that we had previously studied at baseline and at 12 months [6]. In the previous study, we did not have control biopsy specimens from untreated severe asthmatic patients and/or healthy persons, since bronchoscopy and bronchial biopsy are invasive procedures that are not readily applicable in these groups.

Although the number of samples was not large for the usual statistical analysis, the data obtained illustrate a specific trend for both groups of patients (OR and NOR). In particular, our results showed that specimens from OR patients (Gal-3-positive at baseline) confirm the reduction in the thickness of bronchial RBM after 36 months of treatment with anti-IgE, thus emphasizing the possible role of omalizumab in reducing airway remodeling in some cases of persistent allergic asthma [6]. As for molecular components, proteomic analysis showed that mainly ECM, keratin, and smooth muscle proteins were further reduced at 36 months of therapy in Gal-3-positive biopsies.

Although Gal-3 was absent in OR at 36 months, this does not put OR in the same situation as NOR; in fact, OR presented a different proteomic profile, consistent with the reduction in RBM thickness and eosinophil counts. In this context, the lack of Gal-3 in OR at 36 months, combined with the decrease in protein levels reported above, may be correlated with an improvement in remodelling and inflammatory state.

With respect to periostin, Hanania et al. [21] found that patients with high serum periostin had fewer exacerbations after treatment with omalizumab than patients with low serum periostin. This predictive role for periostin was not confirmed in recent clinical trials [22]. In the present study, periostin was not consistently present in the bronchial biopsies of either group, and levels decreased further only in OR after 36 months of treatment. Therefore, based on our data, periostin cannot be regarded as a predictive biomarker of modulation of airway remodeling.

With regard to the bronchial inflammatory pattern, high eosinophil counts in the bronchi have been observed in patients who are eligible for anti-IgE [23] and can be considered one of the biomarkers of the Th2 asthma phenotype. Omalizumab is effective in the treatment of eosinophilic airway inflammation in patients with allergic asthma [24]. In the present study, a further decrease (compared with 12 months) in eosinophils in the biopsy specimens was detected in patients who were Gal-3-positive at baseline (OR), whereas eosinophil counts increased in Gal-3-negative patients (NOR). These findings are consistent with recent data reporting increased and decreased Gal-3 levels in eosinophilic and neutrophilic asthma, respectively [25]. Gal-3 might also predict decreased eosinophilic inflammation in the bronchi in patients receiving omalizumab.

Finally, the further decrease in smooth muscle proteins (i.e., actins and myosins) after 36 months is interesting and thus supports the hypothesis of improved pulmonary function observed in some omalizumab-treated asthmatics [26].

If this was the case, Gal-3 would be a very useful tool for predicting clinical improvement in pulmonary function. This observation is of particular clinical interest, since the above-mentioned studies showed that, as far as FEV_1_ is concerned, not all patients improved with omalizumab. In responsive patients, omalizumab can be considered a disease-modifying treatment and Gal-3 a potential biomarker.

Our findings confirm the role of Gal-3 as a biomarker of long-term reduction in bronchial RBM, eosinophil inflammation, and muscular components in omalizumab-treated severe asthmatics. In addition, our data support the molecular model, indicating that the presence of Gal-3 may have a role in the dissociation of the IgE–FcεRI complex and enhance the effectiveness of anti-IgE therapy for remodelling in Gal-3-positive patients [7]. Further studies based on larger samples or a cellular model are required to confirm the role of Gal-3 in the dissociation of the IgE–FcεRI complex.

Bronchial analysis is useful for investigating the origin and mechanisms of disease; however, in clinical practice, it is necessary to analyze less invasive samples, such as urine and blood. We intend to perform such an analysis in the context of the PROXIMA study (Patient-Reported Outcomes and Xolair^®^ In the Management of Asthma), an observational, multicenter, cross-sectional, prospective cohort study conducted at 25 centers [27]. In fact, PROXIMA involves an ancillary study to explore protein biomarkers in urine and plasma and to characterize them according to severe allergic asthma and treatment effects. In this context, the possible correlation between Gal-3 and evolution of pulmonary function with omalizumab will be investigated in greater depth.

## Conclusions

We investigated the long-term effects of anti-IgE treatment. In particular, proteomic signatures combined with histological data enabled us to confirm the following:

- In bronchial biopsy samples from severe asthmatics, Gal-3 acts as a biomarker of modulation of airway remodeling upon treatment with omalizumab.
- There seems to be a relationship between Gal-3 positivity and improvement in pulmonary function.
- In severe asthmatics treated with omalizumab, Gal-3 is a sufficiently effective biomarker of both short- and long-term reduction in the thickening of bronchial RBM, eosinophilic inflammation, and fibrotic and muscular protein components.

## Additional files

**Additional file 1.** Natural logarithm (ln) of fold change between baseline (T0) and long term anti-IgE treatment (T36) for each subject. Significant values (ln[Fold Change]>|0.6| are are reported in *red* or *blue*: positive (*blue*) and negative (*red*) values indicate increase and decrease at T36, respectively. Specifically, for ln(Fold Change) >0.6 (*blue*) increase at T36; on the contrary, if it is <-0.6 (*red*) decrease at T36. *SMPs*: smooth muscle proteins. ^a^Actual Fold Change was 0 (0/n, or 0/0, see Table 2); for avoiding invalid logarithm it was put at 1/n or 1/1; ^b^Actual Fold Change ∞ (n/0); for avoiding invalid logarithm it was put at n/1. * p-value; T-test2 is without NOR2 subject, because at T36 its behaviour is similar to ORs (se Fig. 6); in bold significant T-tests; ** #/mm2 ratio; *** score ratio.

**Additional file 2.** Complete list of identified proteins in OR (Responders) and NOR (Non-Responders) bronchial biopsies collected after 36 months of anti-IgE treatment. Accession number NCBI, Uniprot entry, Protein name, pI and MW, Spectral Count (SpC) and Score were reported for each protein. The symbol (*) was used to indicate the smooth muscle proteins. The symbol (†) was used to indicate the periostin. The symbol (°) was used to indicate the keratins.

**Additional file 3.** Virtual 2D map in logarithmic scale of FFPE bronchial biopsies, generated using MAProMa software (546 proteins). Proteins are plotted according to their theoretical pI and MW. A color/shape code is assigned to each protein according to SEQUEST score value. Proteins with score ≤15 are reported as *yellow*/*triangle*, proteins with score ≥35 are reported as *red*/*circle*, and proteins in the range 15–35 are reported as *blue*/*square*. The protein lists are reported in Additional file 2.

**Additional file 4.** Changes of abundance levels, calculated by DAVE algorithm from MAPROMA software [9], for eosinoplis, smooth muscle proteins, periostin, keratins and Gal-3 in OR and NOR patients at baseline (T0) and after 36 months (T36) of anti-IgE treatment. Negative value indicates decrease at T36; on the contrary, positive value indicates increase at T36. OR and NOR classification is related to reduction (OR) or not (NOR) of RBM thickness after 12 months of anti-IgE treatment.

**Additional file 5.** Changes of abundance levels, expressed as natural logarithm of score fold change (ln[T36/T0]), for Gal-3 in OR and NOR patients at baseline (T0) and after 36 months (T36) of anti-IgE treatment. Negative value indicates decrease of Gal-3 at T36; on the contrary, positive value indicates increase of Gal-3 at T36 (see Additional file 1). OR and NOR classification is related to reduction (OR) or not (NOR) of RBM thickness after 12 months of anti-IgE treatment.

**Additional file 6.** Differential Analysis (DAVE index) between baseline (T0) and long term anti-IgE treatment (T36) for each subject, using DAVE index from MAPROMA software [9]. Significant values (DAVE > |0.4|) are reported in *red* or *blue*: positive (*blue*) and negative (*red*) values indicate increase and decrease at T36, respectively. *SMPs*: smooth muscle proteins. Of note, DAVE algorithm, tpical of proteomics evaluation, was also applied to eosinophils; the obtained values resulted in good agreement with evaluation obtained by ln[T36/T0] (see also Additional file 1 and compare Fig. 2 and Additional file 4). * p-value; T-test2 is without NOR2 subject, because at T36 its behaviour is similar to ORs (see Fig. 6); in bold significant T-tests.

## Abbreviations

OR: omalizumab responder
NOR: non-omalizumab responder
IgE: immunoglobulin E
MudPIT: multidimensional proteomic identification technology
Gal-3: galectin-3
ECM: extracellular matrix
Th2: T-helper type-2 lymphocytes
FeNO: fractional exhaled nitric oxide
IL: interleukin
pI: isoelectric point
RBM: reticular basement membrane
FEV_1_: forced expiratory volume in the first second
MW: molecular weight
ln: natural logarithm
FC: fold change (ratio T36/T0)
P: p value
