# Supplementary material for: Galectin-3: an early predictive biomarker of modulation of airway remodeling in patients with severe asthma treated with omalizumab for 36 months
Source: Clin Transl Allergy. 2017 Mar 9;7:6. doi: 10.1186/s13601-017-0143-1 (PMC5345272; doi:10.1186/s13601-017-0143-1)
Supplement: Supplementary file 1 — Additional file 1. Natural logarithm (ln) of fold change between baseline (T0) and long term anti-IgE treatment (T36) for each subject. Significant values (ln[Fold Change]>|0.6| are are reported in red or blue: positive (blue) and negative (red) values indicate increase and decrease at T36, respectively. Specifically, for ln(Fold Change) >0.6 (blue) increase at T36; on the contrary, if it is <-0.6 (red) decrease at T36. SMPs: smooth muscle proteins. aActual Fold Change was 0 (0/n, or 0/0, see Table 2); for avoiding invalid logarithm it was put at 1/n or 1/1; bActual Fold Change ∞ (n/0); for avoiding invalid logarithm it was put at n/1. * p-value; T-test2 is without NOR2 subject, because at T36 its behaviour is similar to ORs (se Fig. 6); in bold significant T-tests; ** #/mm2 ratio; *** score ratio. [file 13601_2017_143_MOESM1_ESM.doc]

***Additional file 1 – N****atural logarithm (ln) of Fold Change between baseline (T0) and long term anti-IgE treatment (T36) for each subject.*

*Significant values (ln[Fold Change]>|0.6| are are reported in red or blue: positive (blue) and negative (red) values indicate increase and decrease at T36, respectively. Specifically, for ln(Fold Change) >0.6 (blue) increase at T36; on the contrary, if it is < - 0.6 (red) decrease at T36.*

*SMPs: Smooth Muscle Proteins*

^a^*Actul Fold Change was 0 (0/n, or 0/0, see Table 2); for avoiding invalid logarithm it was put at 1/n or 1/1.*

^b^*Actual Fold Change ∞ (n/0); for avoiding invalid logarithm it was put at n/1.*

** p-value;T-test2 is without NOR2 subject, because at T36 its behaviour is similar to ORs (se Figure 6); in bold significant T-tests.*

*** #/mm^2^ ratio*

**** score ratio*

|  | **ln[T36/T0]** | | | | | | | | | |
| --- | --- | --- | --- | --- | --- | --- | --- | --- | --- | --- |
|  | OR1 | OR2 | OR3 | OR4 | NOR1 | NOR2 | NOR3 | NOR4 | *T-test1 | *T-test2 (without NOR2) |
| Eosinophils** | -1.85 | -1.24 | -2.67 | -3.14 | 4.70^a^ | -1.20 | 0.69 | 1.10 | **0.010** | **0.002** |
| SMPs*** | -3.36 | -1.39 | -5.29 ^b^ | -6.20 ^b^ | 5.12 ^a^ | 0.00 ^a^ | 0.41 | 4.58 ^a^ | **0.050** | **0.008** |
| Periostin*** | -3.40 ^b^ | -5.08 ^b^ | -4.87 ^b^ | -5.01 ^b^ | 3.00 ^a^ | -3.40 ^a^ | -0.12 | 3.00 ^a^ | **0.019** | **0.007** |
| Keratins*** | -1.24 | -1.93 | -2.79 | -2.04 | 0.41 | -3.87 | -0.35 | -0.18 | 0.193 | **0.002** |
| Galectin 3*** | -2.30 | -2.30 | -3.40 | -3.00 | 0.00 | 0.00 | 0.00 | 0.00 | **0.001** | **0.001** |
