# Supplementary material for: Galectin-3: an early predictive biomarker of modulation of airway remodeling in patients with severe asthma treated with omalizumab for 36 months
Source: Clin Transl Allergy. 2017 Mar 9;7:6. doi: 10.1186/s13601-017-0143-1 (PMC5345272; doi:10.1186/s13601-017-0143-1)
Supplement: Supplementary file 3 — Additional file 3. Virtual 2D map in logarithmic scale of FFPE bronchial biopsies, generated using MAProMa software (546 proteins). Proteins are plotted according to their theoretical pI and MW. A color/shape code is assigned to each protein according to SEQUEST score value. Proteins with score ≤15 are reported as yellow/triangle, proteins with score ≥35 are reported as red/circle, and proteins in the range 15–35 are reported as blue/square. The protein lists are reported in Additional file 2. [file 13601_2017_143_MOESM3_ESM.doc]

**Additional file 3** - Virtual 2D map

*
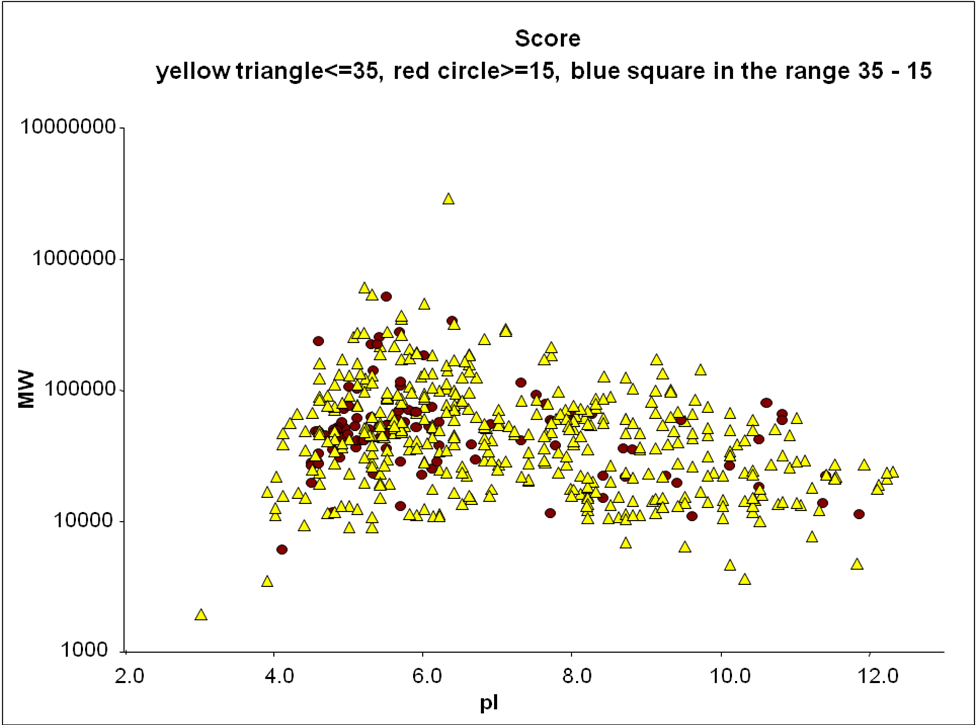
*

**Additional file 3** - Virtual 2D map in logarithmic scale of FFPE bronchial biopsies, generated using MAProMa software (546 proteins). Proteins are plotted according to their theoretical pI and MW. A color/shape code is assigned to each protein according to SEQUEST score value. Proteins with score ≤15 are reported as yellow/triangle, proteins with score ≥35 are reported as red/circle, and proteins in the range 15–35 are reported as blue/square. The protein lists are reported in Additional file 1.
