# Supplementary figures and images for: Galectin-3: an early predictive biomarker of modulation of airway remodeling in patients with severe asthma treated with omalizumab for 36 months
Source: Clin Transl Allergy. 2017 Mar 9;7:6. doi: 10.1186/s13601-017-0143-1 (PMC5345272; doi:10.1186/s13601-017-0143-1)

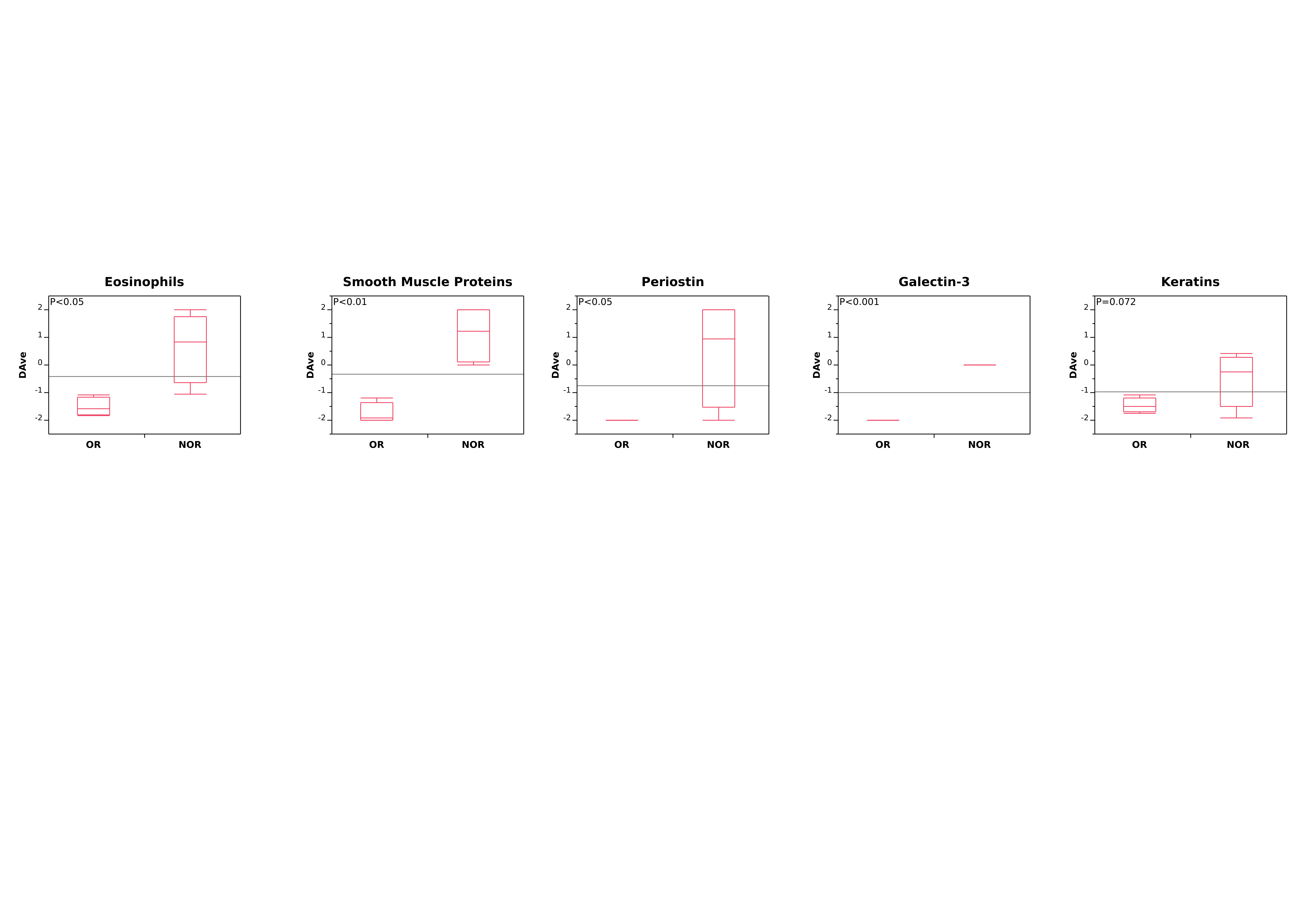

Supplement: Supplementary file 4 — Additional file 4. Changes of abundance levels, calculated by DAVE algorithm from MAPROMA software [9], for eosinoplis, smooth muscle proteins, periostin, keratins and Gal-3 in OR and NOR patients at baseline (T0) and after 36 months (T36) of anti-IgE treatment. Negative value indicates decrease at T36; on the contrary, positive value indicates increase at T36. OR and NOR classification is related to reduction (OR) or not (NOR) of RBM thickness after 12 months of anti-IgE treatment. [file 13601_2017_143_MOESM4_ESM.png]

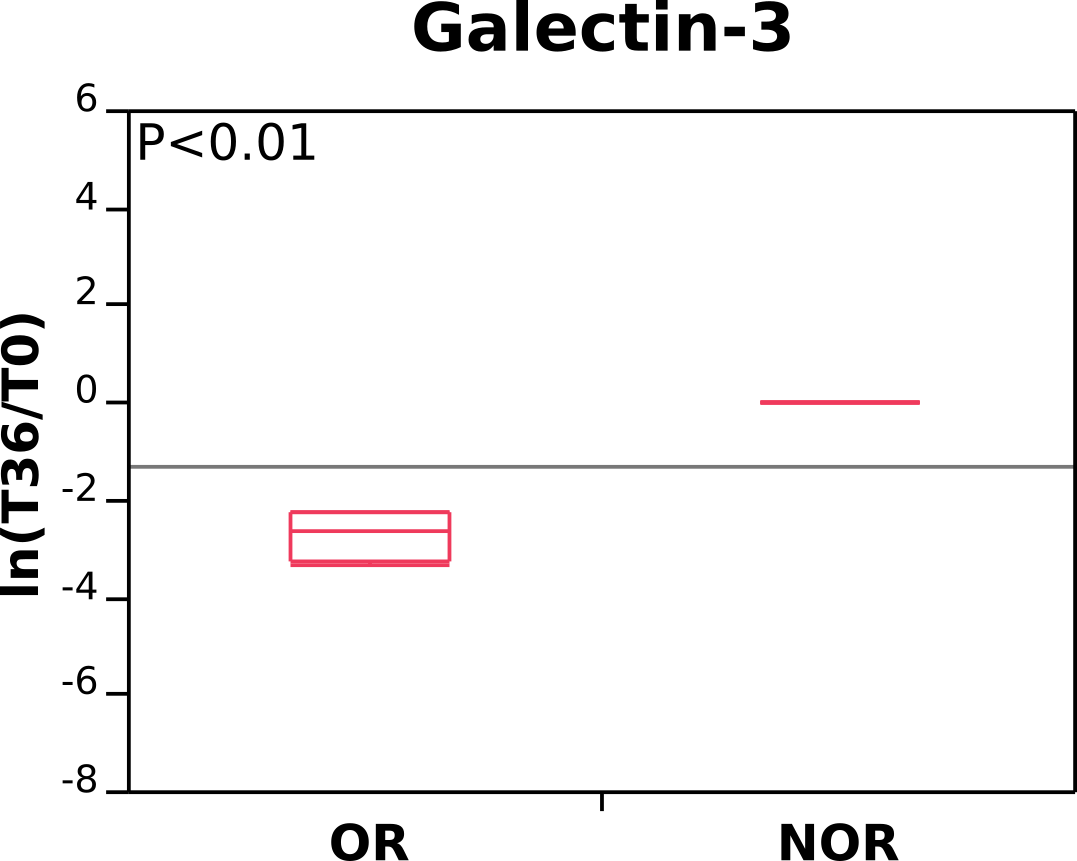

Supplement: Supplementary file 5 — Additional file 5. Changes of abundance levels, expressed as natural logarithm of score fold change (ln[T36/T0]), for Gal-3 in OR and NOR patients at baseline (T0) and after 36 months (T36) of anti-IgE treatment. Negative value indicates decrease of Gal-3 at T36; on the contrary, positive value indicates increase of Gal-3 at T36 (see Additional file 1). OR and NOR classification is related to reduction (OR) or not (NOR) of RBM thickness after 12 months of anti-IgE treatment. [file 13601_2017_143_MOESM5_ESM.png]
