# Supplementary material for: Galectin-3: an early predictive biomarker of modulation of airway remodeling in patients with severe asthma treated with omalizumab for 36 months
Source: Clin Transl Allergy. 2017 Mar 9;7:6. doi: 10.1186/s13601-017-0143-1 (PMC5345272; doi:10.1186/s13601-017-0143-1)
Supplement: Supplementary file 6 — Additional file 6. Differential Analysis (DAVE index) between baseline (T0) and long term anti-IgE treatment (T36) for each subject, using DAVE index from MAPROMA software [9]. Significant values (DAVE > |0.4|) are reported in red or blue: positive (blue) and negative (red) values indicate increase and decrease at T36, respectively. SMPs: smooth muscle proteins. Of note, DAVE algorithm, tpical of proteomics evaluation, was also applied to eosinophils; the obtained values resulted in good agreement with evaluation obtained by ln[T36/T0] (see also Additional file 1 and compare Fig. 2 and Additional file 4). * p-value; T-test2 is without NOR2 subject, because at T36 its behaviour is similar to ORs (see Fig. 6); in bold significant T-tests. [file 13601_2017_143_MOESM6_ESM.doc]

***Additional file 6 -*** *Differential Analysis (DAVE index) between baseline (T0) and long term anti-IgE treatment (T36) for each subject, using DAVE index from MAPROMA software [9]. Significant values (DAVE > |0.4|) are reported in red or blue: positive (blue) and negative (red) values indicate increase and decrease at T36, respectively.*

*SMPs: Smooth Muscle Proteins*

*Of note, DAVE algorithm, tpical of proteomics evaluation, was also applied to eosinophils; the obtained values resulted in good agreement with evaluation obtained by ln[T36/T0] (see also Additional file 1 and compare Figure 2 and Additional file 4)*

** p-value;T-test2 is without NOR2 subject, because at T36 its behaviour is similar to ORs (se Figure 6); in bold significant T-tests.*

|  | **Dave T36 vs T0** | | | | | | | |  |  |
| --- | --- | --- | --- | --- | --- | --- | --- | --- | --- | --- |
|  | **OR1** | **OR2** | **OR3** | **OR4** | **NOR1** | **NOR2** | **NOR3** | **NOR4** | ***T-test** | ****T-test2 (without NOR2)** |
| **Eosinophils (#/mm^2^)** | -1.45 | -1.10 | -1.74 | -1.83 | 2.00 | -1.08 | 0.67 | 1.00 | **0.019** | **0.005** |
| **SMPs** | -1.87 | -1.20 | -2.00 | -2.00 | 2.00 | 0.00 | 0.40 | 2.00 | **0.004** | **0.008** |
| **Periostin** | -2.00 | -2.00 | -2.00 | -2.00 | 2.00 | -2.00 | -0.12 | 2.00 | **0.041** | **0.022** |
| **Keratins** | -1.10 | -1.49 | -1.77 | -1.54 | 0.40 | -1.92 | -0.35 | -0.18 | 0.072 | **0.004** |
| **Galectin-3** | -2.00 | -2.00 | -2.00 | -2.00 | 0.00 | 0.00 | 0.00 | 0.00 | **<0.001** | **<0.001** |
